# Combination of Merocel sponge with Lipopolysaccharide to establish rat rhinosinusitis model

**DOI:** 10.1016/j.bjorl.2023.02.001

**Published:** 2023-02-17

**Authors:** Mengmeng Sun, Junjie Yang, Jiayu Liu, Ming Jing, Leiming Zhang, Wenyu Xin

**Affiliations:** a Key Laboratory of Prescription Effect and Clinical Evaluation of State Administration of Traditional Chinese Medicine of China, School of Pharmacy, Binzhou Medical University, Yantai, Shandong, China; b Key Laboratory of Molecular Pharmacology and Drug Evaluation, School of Pharmacy, Ministry of Education, Yantai University, Yantai, Shandong, China

**Keywords:** Rhinosinusitis, Lipopolysaccharide, Merocel sponge, Animal model

## Abstract

**Objective:**

The study aimed to investigate the feasibility of establishing rhinosinusitis model in rats combinated with Lipopolysaccharide (LPS) and merocel sponge.

**Methods:**

SD (Sprague Dawley) rats that underwent nasal obstruction using Merocel sponge packing, rats with LPS instillation alone, and rats with both nasal obstruction and LPS instillation were used to establish rat models of rhinosinusitis. After the models were established, the nasal symptoms of rats were recorded, the histopathological examination and Transmission Electron Microscopy (TME) of the sinus tissue were performed and the levels of Tumor Necrosis Factor-α (TNF-α), Interleukin-6 (IL-6) in the blood were also analyzed. The expressions of Aquaporin-5 (AQP5), Occludin, Toll-Like Receptor-4 (TLR4), Medullary differentiation factor 88 (MyD88) and phosphorylated (p)-p65 protein were detected by Western blot to evaluate the effect and mechanism of the experimental models.

**Results:**

We found that compared with the control group and LPS group, the sinusitis symptom scores in the Merocel sponge combined with LPS group were significantly increased; the respiratory epithelia of the maxillary sinus were degenerated, cilia were detached, and even inflammatory cell infiltration occurred; the levels of TNF-α and IL-6 were increased; the expression of AQP5 and Occludin protein was decreased; and the expressions of TLR4, MyD88, and p-p65 protein were increased.

**Conclusion:**

For the first time, we successfully established a rat rhinosinusitis model using Merocel sponge with LPS and explored the possible mechanism of LPS action.

## Highlights

• For the first time, a rat rhinosinusitis model was established using LPS and Merocel sponge.
• LPS can induce nasal inflammation through TLR4 signaling pathway.
• LPS can reduce AQP5, occludin protein expression.

## Introduction

Rhinosinusitis is defined as a sudden onset of two or more symptoms, one of which should be either nasal blockage or nasal discharge (anterior or posterior nasal drip). Other symptoms are facial pain or pressure, and impairment or loss of smell. When these symptoms are present for less than 4 weeks, this defines Acute Rhinosinusitis (ARS). When symptoms are present for more than 12 weeks, this represents Chronic Rhinosinusitis (CRS).^1^, ^2^ Rhinosinusitis affects more than 10% of the general population and poses an immense economic burden, accounting for costing more than $11 billion peer year in direct medical expenses.^3^, ^4^

The cause of rhinosinusitis is not entirely clear, however, multiple factors, such as viruses, bacteria, fungi and allergens have been implicated.^2^ The pathophysiology of rhinosinusitis is characterized by congestion of the lining membrane of the nose and paranasal sinuses, associated with increased local infiltration with inflammatory cells and secretion of pro-inflammatory cytokines such as Interleukin-1α (IL-1α), IL-1β, IL-6, IL-8 and Tumor Necrosis Factor-α (TNF-α).^5^, ^6^

In previous studies, rabbits and rats are usually used as modeling objects. The nasal cavity and paranasal sinuses of rabbits are wide, easy to operate, and easy to establish model successfully.^7^, ^8^ However, rhinosinusitis in rabbits is mostly limited to the maxillary sinus, requiring surgical methods to open the maxillary sinus for operation, and rabbits are large and difficult to control.^9^ Rats are simple to perform surgical manipulation and more available reagents and antibodies for studies.^10^ Therefore, we used rats to create the animal model.

Patients with epistaxis or after nasal surgery commonly used the Merocel sponge. It is easy to filled into the nasal cavity and rarely damage the nasal mucosa.^11^ Jin successfully developed an acute bacterial rhinosinusitis mouse model by inserting a sponge containing MRSA COL.^12^ However the bacteria alone could not induce rhinosinusitis, indicating that sponges can achieve bacterial implantation and nasal obstruction.

Lipopolysaccharide (LPS), one of the principal chemical components of the bacterial endotoxin, are located in the outer layer of the Gram-negative bacterial cell wall and consist of three parts: Lipid A, core polysaccharide and O‑specific polysaccharide. In addition to its toxic effects, LPS is able to induce an immune response.^13^, ^14^ When the infection is serious, a large amount of LPS interacts with monocytes and macrophages of the body, resulting in sepsis and septic shock with a high mortality risk.^15^, ^16^ Kim et al. dropped LPS into the nasal cavities of rats in order to establish animal models of rhinosinusitis and 4-days following administration, the sinus mucosa of rats were observed to be significantly thicker.^13^ The aim of this study was to investigate the feasibility of using Merocel sponge as a carrier of LPS to establish a rat rhinosinusitis model. Furthermore, the study planned to analyzed relative protein expressions to further elucidate the role and mechanism of LPS in rhinosinusitis formation.

## Methods

### Animals

The study was conducted with 60 male Sprague Dawley (SD) rats, weighing 180∼200 g, raised and maintained under specific pathogen free conditions. The Animal Ethics Committee of the Binzhou Medical University approved all experimental protocols (Permit Number: 2022-178). All experiments were performed in accordance with the Guide for the Care and Use of Laboratory Animals.

### Establishment of the rhinosinusitis models

**Figure 1:**
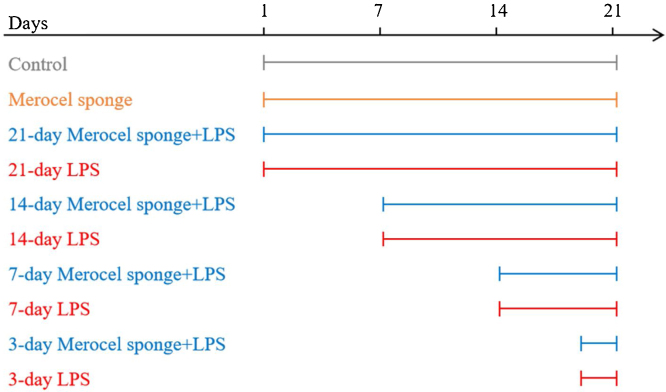
A diagram of the experimental design. Control: normal rats were not treated with Merocel sponge or LPS; Merocel sponge: rats were intranasally packed with Merocel sponge; Merocel sponge + LPS: rats were intranasally packed with Merocel sponge and instilled with LPS; LPS: rats were intranasally instilled with LPS. After LPS instilling, the rats were fed normally for 3, 7, 14 and 21 days.

SD rats were randomly assigned to one of ten groups: control, Merocel sponge, 3-day LPS, 3-day Merocel sponge + LPS, 7-day LPS, 7-day Merocel sponge + LPS, 14-day LPS, 14-day Merocel sponge + LPS, 21-day LPS, 21-day Merocel sponge + LPS. For the control group, 10 μL of sterile normal saline solution was dropped into the nasal cavities. Rats in LPS group received 10 μg of LPS in 10 μL of sterile normal saline solutions. For Merocel sponge group, Merocel sponge inserted into right nasal cavity and then 10 μL of sterile normal saline solution was instilled. For Merocel sponge combined with LPS groups, Merocel sponge inserted into right nasal cavity and then 10 μg of LPS was instilled. Sterile normal saline solutions with or without LPS instilled once a day for three consecutive days. After LPS instilling, the rats were fed normally for 3, 7, 14 and 21 days (Merocel sponge had not been withdrawn) (Fig. 1).

### Nasal symptom score

One hour after LPS instillation, the nasal symptoms of rats were observed every day, and the symptom scores of rats within 30 min were recorded according to the rat rhinosinusitis symptom scoring criteria^17^ (Table 1).

**Table 1:** Score criteria for acute rhinosinusitis symptoms.

Symptoms	Scoring criteria		
	Mild (1-point)	Medium (2-points)	Heavy (3-points)
Number of scratching nose in 30 min	1‒4 times	5‒9 times	More than 10-times
Number of sneezes in 30 min	1‒4	5‒9	More than 10
Nasal discharge	A little runny nose	Runny nose to nostrils	Secretions attached around the nasal cavityvity
Inflammation of the nasal cavity	Nasal congestion	Nasal inflamed	Nasal inflamed and bleedingleeding

## ELISA

The blood was centrifuged at 3000 rpm for 10 min and the supernatant was collected. The concentrations of TNF-α and IL-6 in serum were evaluated by ELISA assay according to the manufacturer's protocol.

### Histopathological analysis and Immunohistochemistry (IHC)

The rats' heads were excised, then fixed in 4% paraformaldehyde for 24 h, decalcified in a rapid decalcifying solution, embedded in a paraffin block. The maxillary sinus and ethmoid sinus were selected for Hematoxylin-Eosin (H&E) to staining, and the inflammatory infiltration of nasal cavity and sinuses was observed by Olympus optical microscope.

For immunohistochemistry, the sections were incubated overnight at 4 °C with primary antibodies for AQP5 and Occludin. And then, sections were incubated with a secondary antibody at room temperature.

### Transmission Electron Microscopy (TEM)

The nasal mucosa was fixed in 2.5% glutaraldehyde, treated with 1% osmic acid, dehydrated with different concentrations of ethanol, replaced with acetone, sliced after embedding, stained, and observed by TEM.

### Western blotting

After removing the nasal mucosa, the total protein was extracted with a lysis buffer containing protease inhibitors. Protein levels in these lysates were quantified with a BCA protein assay kit. A total of 50 μg of protein per sample was then separated via 10% SDS-PAGE prior to transfer onto a PVDF membrane that was blocked for 2 h using 5% non-fat milk and probed overnight with antibodies specific for Aquaporin-5 (AQP5), Medullary Differentiation factor-88 (MyD88), Occludin, p65, phosphorylated (p)-p65, Toll-like Receptor-4 (TLR4) (1:1000) at 4°C. Blots were then probed for 1 h with horseradish peroxidase conjugated secondary antibodies, followed by detection of protein bands via chemiluminescence.

### Statistical analysis

IBM SPSS Statistics 23.0 was used for statistical analysis, and all data expressed as mean ± Standard Deviation (SD). Data were analyzed for statistical significance by the ANOVA that followed the normal distribution and met variance homogeneity. Comparisons between time points, such as sinusitis symptom scores, was analyzed using ANOVA and repeated measures; *p* < 0.05 was considered statistically significant.

## Results

### Merocel sponge packing and LPS stimulation could aggravate the symptoms of rhinosinusitis in rats

**Figure 2:**
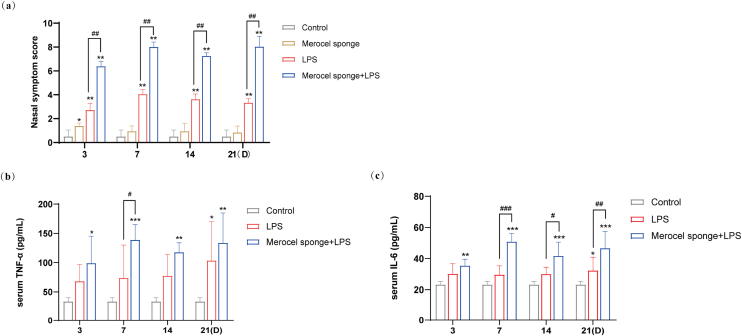
(a) Rat nasal symptom scores within 21 days. TNF-α (b) and IL-6 (c) in the serum blood in rats. Grey columns represent control group; orange columns represent Merocel sponge group; red columns represent LPS group; blue columns represent Merocel sponge combined LPS group. *p < 0.05, **p < 0.01, ***p < 0.001; ^#^*p* < 0.05, ^##^*p* < 0.01, ^###^*p* < 0.001, * vs. control; ^#^ vs. Merocel sponge + LPS.

After the completion of modeling, rats in the experimental group gradually developed sneezing and runny nose symptoms, and during the observation period, the Merocel sponge in the rat's nose did not migrate. The nasal symptoms in rat were observed for 30 min, and the recorded data showed that the scores of nasal symptoms in rats with rhinosinusitis were significantly increased in experimental group as compared with that in control group. Meanwhile, compared with LPS group, the nasal symptom scores in the group combining Merocel sponge with LPS were markedly increased (*p* < 0.01) (Fig. 2a), and the rhinosinusitis symptoms were more obvious.

### Merocel sponge packing and LPS stimulation could aggravate the levels of serum inflammatory cytokines TNF-α and IL-6

The levels of inflammatory cytokines TNF-α and IL-6 in the control group were low. Compared with the control group, the contents of serum inflammatory cytokines TNF-α and IL-6 were observably increased in the group Merocel sponge combined LPS (*p* < 0.05 and *p* < 0.001), while the levels of TNF-α and IL-6 were increased only at 21 days in LPS group (*p* < 0.05).

On day-7, the levels of TNF-α and IL-6 were significantly increased in Merocel sponge combined LPS group compared with LPS group (*p* < 0.05 and *p* < 0.001). On day 14 and 21, the levels of IL-6 were remarkably grew in Merocel sponge combined LPS group compared with LPS group (*p* < 0.05 and *p* < 0.01) (Fig. 2b and c). These results demonstrated that, compared with LPS group, Merocel sponge combined LPS group could aggravate the levels of TNF-α and IL-6 and the inflammatory response.

### Merocel sponge packing and LPS stimulation could aggravate the pathological changes of nasal mucosa and reduce cilia and microvilli in rat models

**Figure 3:**
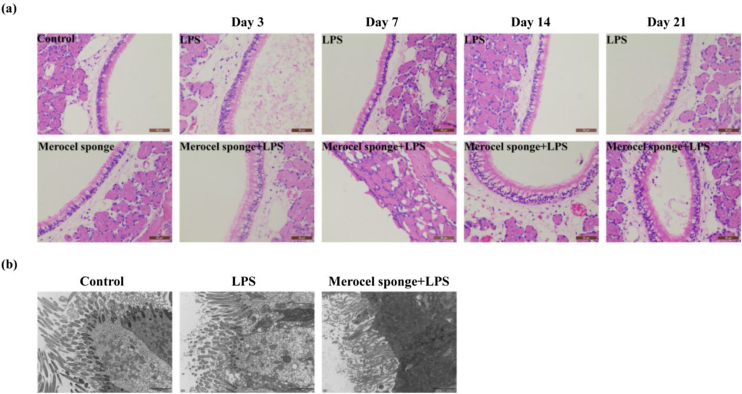
SD rats were divided into control group, Merocel sponge group, LPS group and Merocel sponge combined with LPS group. Rats in the experimental group were fed normally for 3, 7, 14, and 21 days after LPS instillation, and nasal tissues were taken for H&E and TEM. (a) H&E staining diagram of rat nasal mucosa. (b) The nasal mucosa of rats of day 7 was observed by TEM.

To quantify the effect of Merocel sponge packing and LPS stimulation on the degree of pathology in rat models, H&E staining and TEM were performed. The results showed that the respiratory epithelia of the maxillary sinus were intact in the control group. In LPS group and Merocel sponge group, only epithelial cell degeneration was observed, while Merocel sponge combined with LPS group, epithelial cell degeneration was observed on Day-3; severe epithelial cell degeneration, cilia disappearance and cell detachment were observed on Day-7; epithelial cell degeneration and cilia detachment were observed on Day-14; epithelial cell degeneration and cilia detachment and inflammatory cell infiltration were observed on Day-21 (Fig. 3a).

In the control group, the cilia and microvilli were arranged neatly, the organelles were intact, and the intercellular junctions were normal. In the 7-day LPS group, the number of ciliary microvilli was large, the intercellular junction surface was regular, and the organelle structure was basically normal. However, in the 7-day Merocel sponge combined with LPS group, a small number of cilia and microvilli were observed, the intercellular junction surface was irregular and significantly enlarged; the organelle was vacuolated (Fig. 3b).

Therefore, packing with Merocel sponge and instillation of LPS could aggravate nasal mucosal injury in rats.

### LPS stimulation decreases AQP5, Occludin protein expressions in the nasal mucosa of rats

**Figure 4:**
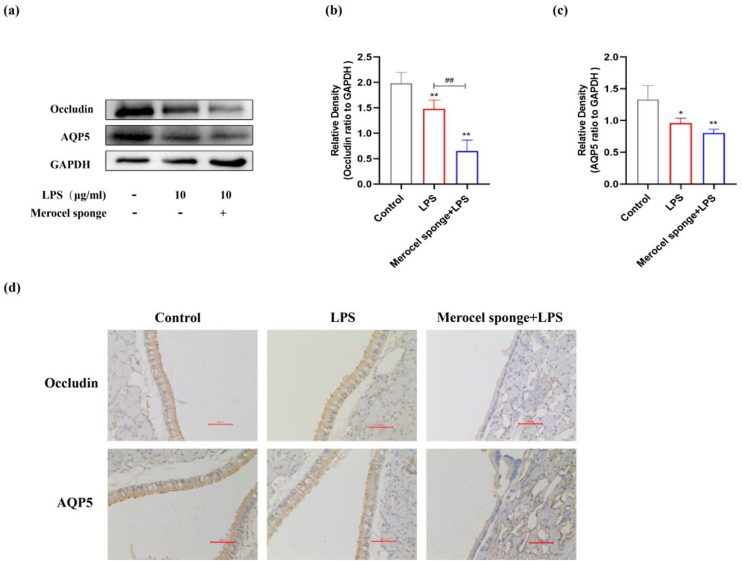
(a) Protein expression of Occludin, AQP5 in the nasal mucosa of rats at 7-days after model establishment. Protein levels of Occludin (b), AQP5 (c). GAPDH was regarded as an internal reference. (d) IHC staining of AQP5 and Occludin in the nasal mucosa of rats. *p < 0.05, **p < 0.01, ^##^*p* < 0.01, * vs. control; ^#^ vs. Merocel sponge + LPS.

The expressions of AQP5 and Occludin protein in the nasal mucosa of rats at 7 days after LPS treatment were investigated. The expressions of AQP5 and Occludin protein were significantly decreased after establishing the model (*p* < 0.05 and *p* < 0.01) (Fig. 4a‒c). Compared with LPS group, Occludin protein expression was significantly decreased in merocel sponge combined with LPS group and AQP5 protein expression was not statistically significant. IHC staining results consistent with the immunoblotting data (Fig. 4d). Therefore, the results indicated that LPS stimulation may decrease the expressions of AQP5 and Occludin protein and the effect was more remarkable in the group combined with Merocel sponge and LPS.

### LPS stimulation activates TLR4/MyD88/nuclear factor κB (NF-κB) signaling pathway in the nasal mucosa of rats

**Figure 5:**
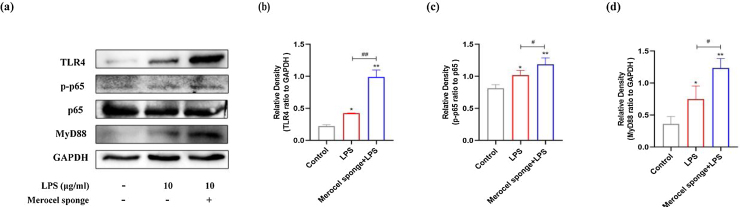
(a) Protein expression in TLR4-related pathway in the nasal mucosa of rats at 7-days after model establishment. Protein levels of TLR4 (b), PP65(c), MyD88 (d). GAPDH was regarded as an internal reference. *p < 0.05, **p < 0.01, ^#^*p* < 0.05, ^##^*p* < 0.01, * vs. control; ^#^ vs. Merocel sponge + LPS.

Western blot analysis revealed that the protein levels of TLR4, MyD88, and pp65 were increased after LPS treatment at day-7 (*p* < 0.05 and *p* < 0.01) (Fig. 5). The expressions of TLR4, MyD88, and pp65 protein were significantly increased in merocel sponge combined with LPS group compared with LPS group (*p* <  0.05 and *p* < 0.01) (Fig. 5). The results indicated that LPS stimulation may activate TLR4/MyD88/NF-κB signaling pathway and the effect was more obvious in the group combined with Merocel sponge and LPS.

## Discussion

Rhinosinusitis is an inflammatory disease that occurs in the paranasal sinuses and nasal passages. Rhinosinusitis has a high incidence and far exceeds other respiratory diseases such as acute asthma and chronic bronchitis.^18^ The establishment of animal models of rhinosinusitis has brought great convenience to the related research of this disease. The experimental animal model established in this study is simple to perform, easy to establish, and has high model stability, and more importantly this new model conforms to the pathophysiological characteristics of human rhinosinusitis.

Irritation of the respiratory tract by toxic gases, air pollutants, bacteria, and viruses can lead to respiratory inflammation. LPS is a major component of endotoxin and is a mixture of cell wall components of Gram-negative bacteria.^19^ It has been shown that Gram-negative bacteria are frequently associated with rhinosinusitis, which may result when prolonged respiratory exposure to sufficient concentrations of LPS in the environment.^14^ Tiboc-Schnell established an experimental rat model of acute rhinosinusitis by intranasal instillation of 5 μg and 10 μg of LPS (from *E. coli*) to Wister rats for seven consecutive days, and found that rats developed nasal congestion and erythema when repeatedly administered LPS for four days.^20^ Wang successfully developed a mouse chronic rhinosinusitis model by administration of sufficient amounts of LPS over a prolonged period of time.^2^

Several animal models of rhinosinusitis have been established. The most common animal model was nasal packing with Merocel sponge followed by instillation of *Staphylococcus aureus*.^12^, ^21^ The study used Merocel sponge to simulate the obstruction of the nose and LPS was used as the source of infection to induce the development of nasal inflammation. In our study, Merocel sponge group and LPS group did not produce significant symptoms of rhinosinusitis based on scoring and histopathological analyses. For Merocel sponge combined with LPS group, it was found that the symptoms of sneezing and scratching nose were more serious. H&E showed that the respiratory epithelium of the maxillary sinus was degenerated, cilia were detached, and inflammatory cell infiltration even occurred on day-21. The structure of nasal mucosa observed by TEM showed that at 7-days after modeling, cilia and microvilli were reduced, the intercellular junction surface was irregular and significantly enlarged, and organelles were vacuolated. Therefore, Merocel sponges which can induce nasal obstruction are carriers for LPS to take effect.

As one of the first defense systems in contact with the outside world, the nasal cavity can block a large number of pollutants, pathogenic microorganisms and allergens from the body.^22^ Tight Junctions (TJs) between epithelial cells of the nasal mucosa maintain the integrity of the nasal epithelium.^23^ TJs include the claudin family of transmembrane proteins, Occludin, junctional adhesion molecules, and adaptor proteins.^24^ Occludin is widely distributed in human tissues, which expression is often linked to various tissue barrier properties.^25^ AQPs are a group of cell membrane transporters related to water transport. AQPs 1–5 are expressed in human nasal mucosa, but AQP5 is only found in the apical site of epithelial cells and acinar cells. It has been found that AQP5 expression was significantly decreased compared with the normal control group after establishing a LPS-induced acute lung injury model.^26^ It has been reported that AQP5 plays an important role in maintaining nasal fluid balance.^27^ Alan found a significant decrease in AQP5 protein expression in CRS patients with nasal polyps compared to normal patients.^27^ The study showed that AQP5 and Occludin protein expressions were significantly decreased in Merocel sponge combined with LPS group compared with LPS group at day-7. The results showed that LPS stimulation affects barrier function and water transport, taking a greater effect on Merocel sponge combined with LPS group.

LPS is a specific agonist of the inflammatory pathway TLR4 and does not agonize other TLRs.^28^ TLR4 gene deletion or mutation can lead to severe loss of cellular responsiveness to LPS.^29^ Investigations have demonstrated that inappropriate regulation of TLR2 and TLR4 signaling caused inflammatory responses in CRS.^30^, ^31^ The LPS/TLR4 signaling pathway is divided into two transduction pathways, the one is MyD88-dependent response pathway, which mediates the release of proinflammatory cytokines IL-1β, IL-6, IL-8 and TNF-α; the second is non-MyD88-dependent response pathway, which activates NF-κB and mediates the secretion of type I interferon.^32^ In our study, we showed that the levels of TNF-α and IL-6 were increased in experimental groups compared with the control group, and our findings were consistent with other studies conducted previously.^20^ In addition, we found that compared with LPS group, the levels of TNF-α and IL-6 and the expressions of TLR4, MyD88, and pp65 protein were significantly increased in Merocel sponge combined with LPS group. The results showed that LPS induced the production of sinus mucosal inflammation through TLR4-MyD88-NF-κB signaling pathway and the effect was more significant in Merocel sponge combined with LPS group.

In conclusion, in this study, we successfully established a rhinosinusitis model in SD rats using LPS and Merocel sponge. After modeling, the symptoms of sneezing and scratching the nose of rats were obvious, and obvious sinusitis characteristics were observed according to H&E and TME observation. Merocel sponge can simulate the nasal obstruction. LPS can induce the production of sinus mucosal inflammation through TLR4-MyD88-NF-κB signaling pathway. At the same time, LPS stimulation can affect AQP5 and Occludin protein expressions and barrier function and water transport.

## Funding

This study was supported by the National Science Foundation of China (nº 81803546), Key R&D Program of Shandong Province (nº 2019JZZY011104) and the 10.13039/501100007129Natural Science Foundation of Shandong Province (nº ZR2018LH024) (nº ZR2017MH068).

## Conflicts of interest

The authors declare no have conflicts of interest.
